# Diffuse optical spectroscopic method for tissue and body composition assessment

**DOI:** 10.1117/1.JBO.27.6.065002

**Published:** 2022-06-08

**Authors:** Robert V. Warren, Ronen Bar-Yoseph, Brian Hill, Drew Reilly, Abraham Chiu, Shlomit Radom-Aizik, Dan M. Cooper, Bruce J. Tromberg

**Affiliations:** a University of California, Beckman Laser Institute and Medical Clinic, Irvine, California, United States; b University of California, Pediatric Exercise and Genomic Research Center, Irvine, California, United States; c Pediatric Pulmonology Institute, Ruth Rappaport Children’s Hospital, Rambam Health Care Campus, Haifa, Israel; d National Institutes of Health, Eunice Kennedy Shriver National Institute of Child Health and Human Development, Bethesda, Maryland, United States; e University of California, Institute of Clinical Translational Science and Pediatric Exercise and Genomics Research Center, School of Medicine, Department of Pediatrics, Irvine, California, United States; f National Institutes of Health, National Institute of Biomedical Imaging and Bioengineering, Bethesda, Maryland, United States

**Keywords:** near-infrared spectroscopy, diffuse optical spectroscopic imaging, body composition, dual energy x-ray absorptiometry, bed-side monitoring

## Abstract

**Significance:**

Growing levels of obesity and metabolic syndrome have driven demand for more advanced forms of body composition assessment. While various techniques exist to measure body composition, devices are typically expensive and not portable, involve radiation [in the case of dual-energy x-ray absorptiometry (DXA)], and are limited to analysis of adiposity while metabolic information from blood supply and oxygenation are not considered.

**Aim:**

We evaluate whether diffuse optical spectroscopic imaging (DOSI) can be used to predict site-specific adiposity and percent fat (whole body) while simultaneously providing information about local tissue hemoglobin levels and oxygenation.

**Approach:**

DOSI measures of tissue composition in gastrocnemius, quadriceps, abdomen, and biceps, DXA whole-body composition, and ultrasound-derived skin and adipose tissue thickness (SATT) in the quadriceps were obtained from 99 individuals aged 7 to 34 years old.

**Results:**

Various DOSI-derived parameters were correlated with SATT and an optical method is proposed for estimating SATT using a newly defined parameter, the optical fat fraction (OFF), which considers all parameters that correlate with SATT. Broadband absorption and scattering spectra from study participants with the thinnest (SATT≈0.25±0.02  cm) and thickest SATT (SATT≈1.55±0.14  cm), representing best estimates for pure *in vivo* lean and fatty tissue, respectively, are reported. Finally, a trained prediction model is developed which allows DOSI assessment of OFF to predict DXA body-fat percentage, demonstrating that DOSI can be used to quantify body composition.

**Conclusions:**

This study shows that DOSI can be used to assess the adiposity of specific tissues or the entire human body, and the OFF parameter is defined for corroboration and further evaluation in future research.

## 1 Introduction

For several decades, the prevalence of obesity throughout the world has risen with many adverse health effects including diabetes, cardiovascular disease, and metabolic syndromes.^1^^–^^4^ In addition, significant healthcare costs are required to manage obesity-related chronic metabolic diseases.^5^^,^^6^ A statement released by the American Heart Association in 2011 reported that the prevalence of obesity has reached “epidemic/pandemic proportions” and the clinical importance of assessments of adiposity was emphasized.^7^ There is a growing demand for the development of enhanced screening and monitoring techniques for rapid assessment of body composition and metabolism and such technologies will play an important role in developing and optimizing much-needed prevention and treatment strategies.

Body mass index (BMI) has been used for over 100 years to infer body composition, but it is an inaccurate estimate of an individual’s adiposity and primarily works for epidemiologic purposes.^7^^,^^8^ Direct adiposity assessment typically involves cadaver analysis, but indirect methods have been developed that involve various assumptions about biological tissue, such as hydrodensitometry, magnetic resonance imaging, and dual-energy x-ray absorptiometry (DXA). Direct and indirect methods used to determine adiposity are relatively expensive, typically not portable, and inaccessible by large portions of the population. In addition, there is a need for methods that provide metabolic information from specific regions of the body rather than simply as a whole.^8^ This type of assessment could have utility in the future as evidence continues to emerge that monitoring fat mass and lean mass in specific tissue compartments can reveal important information on disease progression and risk.^8^ In addition, there is likely to be value in readily accessible, accurate, and non-invasive metrics that can be easily repeated for clinical trials and for following children and adolescents during critical periods of growth.

Noninvasive optical absorbance spectroscopy to estimate tissue level oxyhemoglobin obviated the need for painful manual sampling of arterial blood to assess oxygenation and transformed clinical measurement of respiratory status. We hypothesized that near-infrared spectroscopy (NIRS) devices, approximately spanning 600 to 1100 nm, could also present a non-invasive and ionizing radiation-free method to quantify tissue composition and metabolism.^9^^–^^11^ For the assessment of pulse or tissue oxygenation, NIRS devices utilize the absorbance spectrum of hemoglobin and its dependence on oxygenation state.^12^ NIRS can also be used to assess levels of fat and water since these two components absorb uniquely in the NIR spectrum.^13^ Not surprisingly, there are some efforts to develop and commercialize NIRS approaches to measure the key elements of body composition.^14^^,^^15^ As suggested in animal and human studies,^16^^,^^17^ current commercial efforts are, however, limited due to their reliance on continuous-wave NIRS and their dependence on substantial assumptions about tissue scattering, optical pathlength, and composition. Advancements in time- and frequency-domain diffuse optical methods, in combination with broadband time-independent NIRS, has reduced reliance on many of these assumptions^18^ and allowed for multiple physiological variables to be assessed with a single multi-modality device.^10^

In previous work, we utilized an advanced multi-modal NIRS technology, diffuse optical spectroscopic imaging (DOSI), to demonstrate a relationship between optical measures of heme protein, water, and fat content with DXA measures of lean soft tissue.^13^ This finding provided evidence that DOSI could be used to non-invasively and quantitatively measure body composition in specific tissue compartments. In addition, we have also used DOSI to monitor adipose tissue of adult obese individuals as they underwent caloric restriction and reported that absorption and scattering changes track and may elucidate metabolic changes accompanying weight loss.^9^ These two studies were limited to the gastrocnemius and the abdomen in adults, respectively.

In this work, we used DOSI, ultrasound (US), and DXA to characterize local tissue and whole-body composition in 99 healthy, non-obese participants aged 7 to 34 years. Our DOSI protocol quantifies hemo+myoglobin, water, fat, and the wavelength dependence of scattering in several general anatomical regions (gastrocnemius, quadriceps, abdomen, and biceps). In a subset of measurement locations, US was also used to measure skin+adipose tissue thickness (SATT). A model is developed that correlates US with DOSI in order to predict adipose thickness and the properties of fat and muscle using only broadband DOSI data. We propose two new metrics, the optical fat fraction (OFF) and optical lean fraction (OLF), which use DOSI data to describe the adiposity or leanness of a given tissue. Finally, whole-body DXA scans were performed in order to quantify body fat mass and lean soft tissue mass. DXA parameters were used to develop a method for the prediction of whole-body fat and lean soft tissue percentage solely from DOSI data obtained from selected anatomical regions. Our results suggest that portable, diffuse optical technologies can be developed that quantitatively assess both body composition and parameters related to metabolism, such as hemo/myoglobin concentrations and oxygenation. These methods may serve useful tools in the clinical characterization and management of obesity and metabolic disease.

## 2 Methods

### 2.1 Participants and Experimental Design

The study was approved by the UC Irvine Institutional Review Board. Inclusion criteria included healthy 7- to 35-year-old participants without any known respiratory, cardiac, or metabolic disease, and not taking any chronic prescribed medication. BMI of each participant was between the 3rd to 96th percentile for children (Tanner stages 1 to 5) and in the range of 18.6 to 28.2 for adults. Each volunteer visited the Institute for Clinical and Translational Science (ICTS), University of California, Irvine, on six occasions to complete different exercise protocols on cycle ergometer and treadmill. During the first visit, informed consent was obtained (parental consent+child assent for participants <18  years old), demographic and anthropometric data were recorded, and DXA, US, and DOSI were performed prior to any exercise activity. Due to technical limitations, some measurements were collected during other visits. A total of 113 participants were recruited for the study, with 100 completing all DOSI, DXA, and US scanning. Data of one participant were removed due to technical issues with the DOSI measurement, resulting in complete data from 99 participants (Table 1).

**Table 1:** Participant characteristics.

	Age (years)	Height (cm)	Body mass (kg)	BMI	DXA fat%	DXA LST%
Male (n=46)	16.0±6.8 (7.2 to 34.5)	161±18* (125 to 189)	54.6±19.9* (23.0 to 89.8)	20.1±3.8 (13.9 to 28.2)	21.1±6.9* (10.3 to 39.0)	72.7±7.4 (58.2 to 85.6)
Female (n=53)	15.6±6.7 (7.2 to 33.4)	151±15* (113 to 171)	47.2±14.5* (19.7 to 79.9)	19.9±3.3 (14.2 to 27.8)	28.3±5.1* (18.3 to 39.8)	71.0±6.5 (57.7 to 86.1)
Training (n=79)	15.6±6.8 (7.2 to 34.5)	155±17 (125 to 189)	49.5±17.2 (23.0 to 88.5)	19.8±3.4 (14.2 to 28.2)	25.2±6.8 (12.7 to 39.8)	71.9±7.2 (57.7 to 86.1)
Test (n=20)	16.7±6.6 (7.2 to 31.5)	158±18 (113 to 182)	54.8±18.6 (19.7 to 89.8)	21.0±4.0 (13.9 to 27.8)	23.9±7.7 (10.3 to 39.0)	71.4±6.0 (63.0 to 85.6)

Note: A summary of demographics is shown for the entire cohort with two sub-cohort splits. In the top two rows, data from male and female participants are shown. In the bottom two rows, data from the “training” and “test” participants are shown. Statistical differences (*) were detected between males and females in height, body mass, and DXA fat% (p>0.05). No statistical differences were detected between the training and test data sub-cohorts which were used later in the study. n=sample size. Data shown is mean±standard deviation (minimum−maximum).

### 2.2 Diffuse Optical Spectroscopic Imaging Measurements

DOSI measurements were performed using an approach that combines temporally resolved, broadband, frequency-domain measurements with time-independent broadband spectroscopy in a scanning handpiece with source-detector separation of 28 mm. This method and related instrumentation have been extensively described in recent clinical studies.^9^^,^^19^^,^^20^ Briefly, three intensity-modulated laser diodes (690, 785, and 835 nm) and an avalanche photodiode are used to acquire multi-frequency phase and amplitude data. These measurements are used to determine absorption ($\mu_{a}$) and reduced scattering ($\mu_{s}^{\prime }$) coefficients at each wavelength. The scattering data are fit to expected scattering behavior in biological tissue (Mie theory) in the form, which is given by

$$\mu_{s}^{\prime }(\lambda )=A_{500}\times {\left(\frac{\lambda }{500\;\mathrm{nm}}\right)}^{-b},$$ (1)

where $A_{500}$ is the scattering amplitude at 500 nm and b is the scattering power.^9^ Once $A_{500}$ and b have been determined, this function allows for the calculation of reduced scattering coefficients at specific wavelengths of interest. For example, $\mu_{s}^{\prime }(800\;\mathrm{nm})$ would give the reduced scattering coefficient near the isosbestic point of hemoglobin and $\mu_{s}^{\prime }(500\;\mathrm{nm})$ is equivalent to $A_{500}$. In conjunction, a broadband (650 to 1000 nm) continuous-wave (CW) diffuse reflectance spectroscopy measurement is performed using a quartz tungsten halogen lamp and a miniature spectrometer. The broadband reduced scattering results can be used as previously described to determine broadband absorption coefficients from the CW reflectance results.^21^ These absorption coefficients can be further decomposed into basis NIR absorbers. The dominant NIR absorbers in biological tissue are oxygenated hemo+myoglobin (${\mathrm{HbMbO}}_{2}$), de-oxygenated hemo+myoglobin (HbMbR), water, and fat. The sum of ${\mathrm{HbMbO}}_{2}$ and HbMbR concentrations is referred to as the total hemo + myoglobin concentration (THbMb). The percentage of hemo+myoglobin bound to oxygen is known as the tissue oxygen saturation (${\mathrm{StO}}_{2}={\mathrm{HbMbO}}_{2}/\mathrm{THbMb}$). In addition to absorbing chromophores, we chose to include several above-mentioned scattering parameters into our analysis such that our seven optical parameters of interest were: THbMb (μMolar), ${\mathrm{StO}}_{2}$ (%), water (%), fat (%), $A_{500}$ (${\mathrm{mm}}^{-1}$), b (unitless), and $\mu_{s}^{\prime }(800\;\mathrm{nm})$ (units of ${\mathrm{mm}}^{-1}$). While the scattering amplitude at 500 nm is useful to compare to other literature values, we chose to include $\mu_{s}^{\prime }(800\;\mathrm{nm})$ for two additional reasons: (a) 800 nm is in the middle of the NIR window of 600 to 1000 nm, which is most commonly used for deep-tissue diffuse optical spectroscopy, and (b) 800 nm is approximately the isosbestic point of hemoglobin, and reduced scattering coefficients of biological tissue at this wavelength will be of practical use to other researchers.

**Fig. 1:**
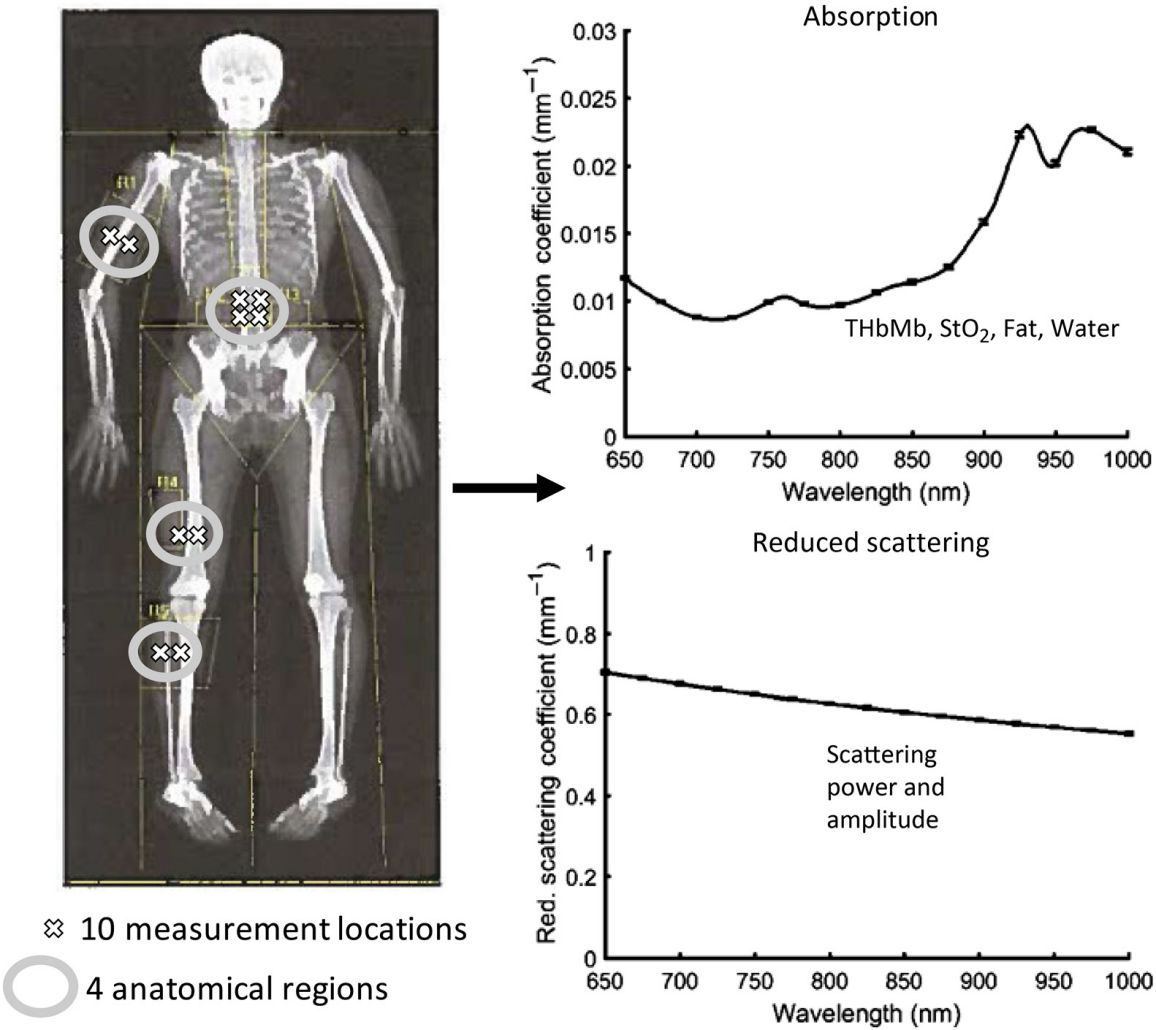
Broadband DOSI data were acquired from 10 specific measurement locations designated by (X) on a representative DXA image (left). Each DOSI measurement contains broadband, 650 to 1000 nm absorption and reduced scattering coefficients (shown to the right for an abdominal measurement). At each specific location, spectral data are decomposed into constituent absorbing chromophores and scattering features, namely: THbMb, ${\mathrm{StO}}_{2}$, fat, water, scattering power, and scattering amplitude. Prior to comparison with DXA, DOSI constituent data from specific measurement locations (Xs) were averaged into four general anatomical regions (marked with gray ovals) for comparison with DXA (biceps, abdomen, quadriceps, and calf). DOSI, diffuse optical spectroscopic imaging; DXA, dual-energy x-ray absorptiometry; THbMb, tissue total hemo+myoglobin concentration; ${\mathrm{StO}}_{2}$, oxygen saturation of tissue hemo+myoglobin; water, tissue water content; fat, tissue fat content.

Ten anatomical sites were chosen and measured with DOSI for each participant (Fig. 1). These 10 measurement locations were the short- and long-heads of the biceps (BS and BL), four locations surrounding the navel on the abdomen (LL, LR, UL, and UR), the vastus lateralus (VL), rectus femoris (RF), and medial and lateral gastrocnemius (GM and GL). At each location, three DOSI measurements were performed, and results were averaged together to reduce the potential for measurement error. We note that a previous study by Cerussi et al. found that DOSI measurements had “<5% variance over the course of several instruments, operators, phantoms, and time points (∼8  months).” While we performed an abundance of measurements in close proximity to each other (e.g., GM and GL within 10 cm of each other, LL, LR, UL, and UR are within 10 cm of each other), we did this partially for redundancy and also in an attempt to further average and reduce measurement error. Rather than keep all measurement locations during analysis, results from certain anatomical regions were averaged together so only four anatomical regions remained. We also did this since repeat measures on participants can falsely inflate correlation coefficients and lead to overfitting. In particular, BS and BL were averaged together to produce a single result for the biceps, LL, LR, UL, and UR were averaged together to produce a single result for the abdomen, VL and RF were averaged together to produce a single result for the quadriceps, and GM and GL were averaged together to produce a single result for the calf. These easily accessible positions were chosen for two primary reasons: (1) to sample a range of the human body spanning from the upper limbs through the torso and into the lower legs and (2) because participants did not need to entirely remove clothing for the measurements.

### 2.3 Ultrasound Measurements

**Fig. 2:**
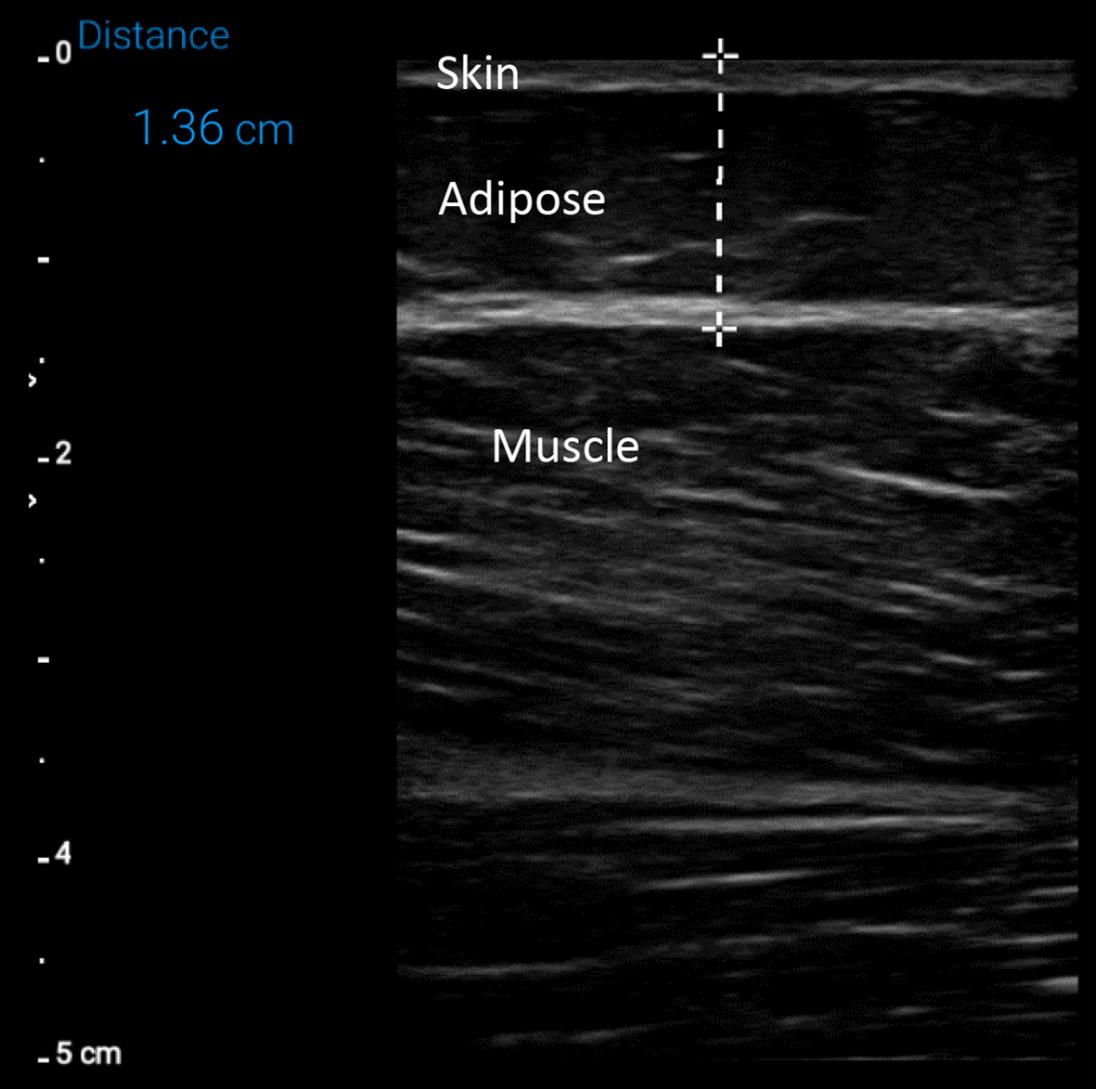
A sample ultrasound image that was used to measure SATT. In each ultrasound image, the outermost layer was skin, followed by an intermediate subcutaneous adipose tissue layer, followed by the muscle layer. SATT was determined as the distance between the outermost surface of the skin and the outermost surface of the muscle.

US measurements of SATT were performed using a portable US device (Lumify, Phillips) with a broadband linear array transducer (Lumify L 12-4). The same operator performed measurements of SATT over the RF muscle and the VL muscle. SATT thickness was measured from the superficial layer of the skin to the superficial layer of the muscle (Fig. 2). The RF and VL measurements were performed in the same location as DOSI measurements, which were approximately the lower third of the anterolateral part of the thigh.

### 2.4 Dual-Energy X-Ray Absorptiometry Measurements

DXA measurements were performed using a Hologic Discovery A DXA system with Apex 3.3 software (Hologic, Marlborough, Massachusetts, United States). Prior to scanning, participants removed all metal objects and lay supine on a padded table. Using the standard approach with DXA, a three-component model was utilized to quantify fat mass, lean soft tissue mass, and bone mineral content for each participant,^22^ and these parameters were converted to fat percentage (fat%), lean soft tissue percentage (LST%), and bone (bone%), respectively, by dividing by total body mass, and the related equations are

Total body mass=Fat mass+Lean soft tissue mass+Bone mass, (2)

$$\text{Fat}\%=\frac{\text{Fat mass}}{\text{Fat mass}+\text{Lean soft tissue mass}+\text{Bone mass}},$$ (3)

$$\mathrm{LST}\%=\frac{\text{Lean soft tissue mass}}{\text{Fat mass}+\text{Lean soft tissue mass}+\text{Bone mass}},$$ (4)

and

$$\text{Bone}\%=\frac{\text{Bone mass}}{\text{Fat mass}+\text{Lean soft tissue mass}+\text{Bone mass}}.$$ (5)

Only fat% and LST% were further analyzed in this study, as we did not expect DOSI to be sensitive to bone mineral content given the locations of our measurements and the probable penetration depths of NIR light.

### 2.5 Fat and Lean Tissue Distinction by DOSI

Data from SATT measurements were sorted to identify the five participants with the thickest SATT measurements (SATT≈1.55±0.14  cm) and five participants with the thinnest SATT measurements (SATT≈0.25±0.02  cm). DOSI data were further analyzed from these sub-cohorts to isolate optical properties (absorption and reduced scattering coefficients) and optical chromophore concentrations. We hypothesized that these two sets of data approximate when the signal is predominantly from adipose tissue (thickest SATT) or when the signal is predominantly from muscle tissue (thinnest SATT).

### 2.6 Statistical Analysis

All data analysis and statistical tests were performed within MATLAB (2021b, The MathWorks, Inc.) and were derived from the *Handbook of Biological Statistics* (McDonald 2014). Statistical significance is defined as α<0.05 throughout this study. In addition, whenever multiple hypotheses were tested in a similar class of comparisons a Bonferroni correction was applied to determine a test-specific p-value which would meet our desired significance criteria of α<0.05. These instances of multiple hypothesis testing are noted later in this section.

For participant demographics, a two-sample t-test (MATLAB’s t-test2) was used to test the null hypothesis that sub-cohort samples (e.g., male versus female, training versus test) came from populations with equal means and equal variances.

The goal for DOSI and US comparisons was to determine which DOSI parameters were associated with SATT prior to the development of a prediction method. Results were available from two measurement locations in the quadriceps for each participant (RF and VL). As repeat measures on participants can falsely inflate correlation coefficients, data from the two measurement locations were averaged together prior to correlation tests. After viewing scatter plots of data and identifying non-linear relationships, Spearman rank correlation coefficients were calculated between seven individual DOSI parameters and SATT (p<0.0083 for significance). This initial set of correlation tests was used to determine if specific parameters of DOSI data were significantly associated with SATT. In addition to meeting the correlation significance criteria, we required that squared correlation coefficients be greater than ($R^{2}>0.5$) if they would be considered in our proposed prediction method. DOSI parameters which met the significance criteria and explained a large amount of variation in SATT were combined into a fractional metric, the OFF, by placing positive correlates of SATT in the numerator and all correlates in the denominator. Similarly, an alternative metric, the OLF, is defined where negative correlates of SATT are placed in the numerator and all correlates are placed in the denominator. For the prediction method, a functional relationship was determined between the OFF and SATT by using the Curve Fitting app in MATLAB. A randomly assigned training set of 79 participants (80%) was used for curve fitting and a test set of the remaining 20 participants (20%) was used to evaluate the accuracy of the predictive method.

The goal for DOSI and DXA comparisons was to build a regression model for prediction of DXA results given DOSI results at the four general anatomical regions (biceps, abdomen, quadriceps, and calf). During comparisons with SATT, we determined the OFF and the OLF from DOSI and these parameters were used when comparing to DXA. After viewing scatter plots of DOSI and DXA data and identifying linear relationships, Pearson product-moment correlation coefficients were calculated between DXA fat% and OFF and DXA LST% and OLF at each of four general anatomical regions (four tests in total per DXA parameter). This initial set of correlation tests was used to determine if DOSI data taken from general anatomical regions were significantly associated with a corresponding parameter from DXA (p<0.0125). Similar to the correlation tests with SATT, we also determined if DOSI parameters explained a large degree of variation in DXA parameters ($R^{2}>0.5$). If correlations were not significant or did not explain a large degree of DXA variation at a given measurement location, this DOSI parameter was not considered for our final regression model.

A randomly assigned training set of 79 participants (80%) was used for regression modeling and a test set of the remaining 20 participants (20%) was used to evaluate the accuracy of the regression model. A generalized linear model was used for regression in MATLAB (glmfit) where OFF or OLF from all four general anatomical regions were used as predictors of DXA fat% or LST%, respectively.

## 3 Results

### 3.1 Participant Demographics

In all, 99 participants (53 female, 46 male) aged 7 to 34 years completed body composition assessments with DOSI, US, and DXA (Table 1). While age, BMI, and DXA LST% were closely matched between sexes, height, body mass, and DXA fat% were different between sexes (α<0.05). All anthropometric characteristics were closely matched between the training and test sub-cohorts that are used later in the study.

### 3.2 DOSI and Skin + Adipose Tissue Thickness

**Fig. 3:**
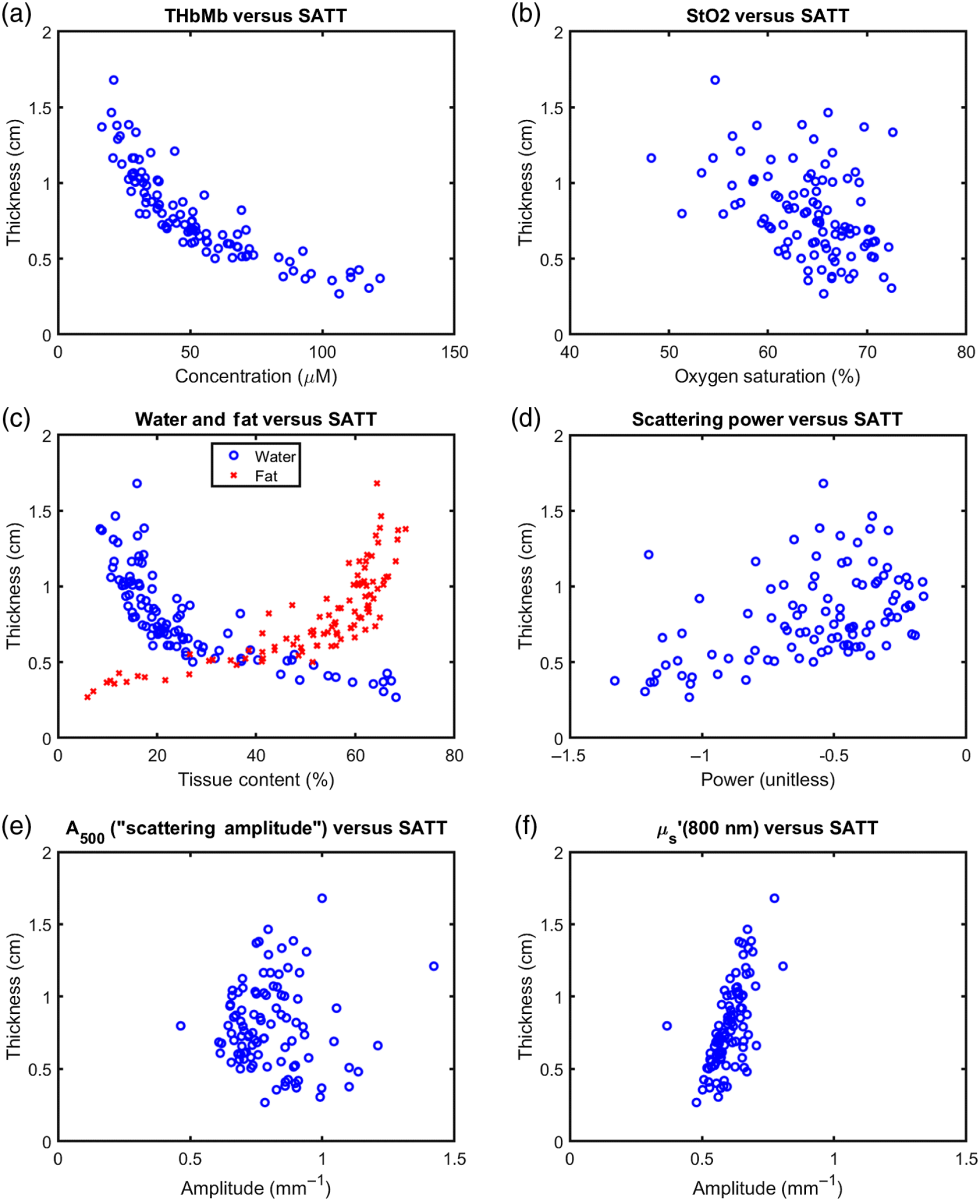
Comparison of DOSI results in the quadriceps (mean of rectus femoris and vastus lateralus results) with the skin+adipose tissue thickness as obtained from ultrasound. (a) THbMb, (b) ${\mathrm{StO}}_{2}$, (c) water, (d) fat, (e) scattering power, and (f) $\mu_{s}^{\prime }(800\;\mathrm{nm})$ were significantly correlated with SATT (p<0.0083) while (e) $A_{500}$ (“scattering amplitude”) was not. However, only THbMb, water, and fat resulted in $R^{2}>0.5$.

After averaging the results from the RF and VL measurement locations into a single anatomical region (quadriceps), significant correlations were detected between SATT and all DOSI parameters except $A_{500}$ (Fig. 3 and Table 2). In addition, correlation coefficients >0.5 were only detected between SATT and THbMb, water, and fat (Table 2). Since fat was positively correlated with SATT and THbMb and water were negatively correlated with SATT, we define

$$\mathrm{OFF}=\frac{\text{Fat}}{\mathrm{THbMb}+\text{Water}+\text{Fat}},$$ (6)

and

$$\mathrm{OLF}\;=\;\frac{\mathrm{THbMb}\;+\;\mathrm{Water}}{\mathrm{THbMb}\;+\;\mathrm{Water}\;+\;\mathrm{Fat}}.$$ (7)

**Table 2:** Correlation coefficients and p-values for Spearman correlation tests between DOSI parameters and ultrasound SATT. While all DOSI parameters except scattering amplitude met the significance criteria for this study, only THbMb, water, and fat resulted in $R^{2}\text{ values}>0.5$.

DOSI parameter	$R^{2}$	p
THbMb	0.87	8.6e-45
${\mathrm{StO}}_{2}$	0.17	2.0e-5
Water	0.80	6.1e-36
Fat	0.79	5.7e-35
$A_{500}$	0.00	0.53
b	0.25	1.4e-7
$\mu_{s}^{\prime }(800\;\mathrm{nm})$	0.46	1.6e-14

It is important to note that these fractions contain mixed units and are only convenient since fat and water are in units of % (values 0 to 100) and THbMb is in units of μM (which also has values in the range of 0 to ∼200 in mixed biological tissue).

**Fig. 4:**
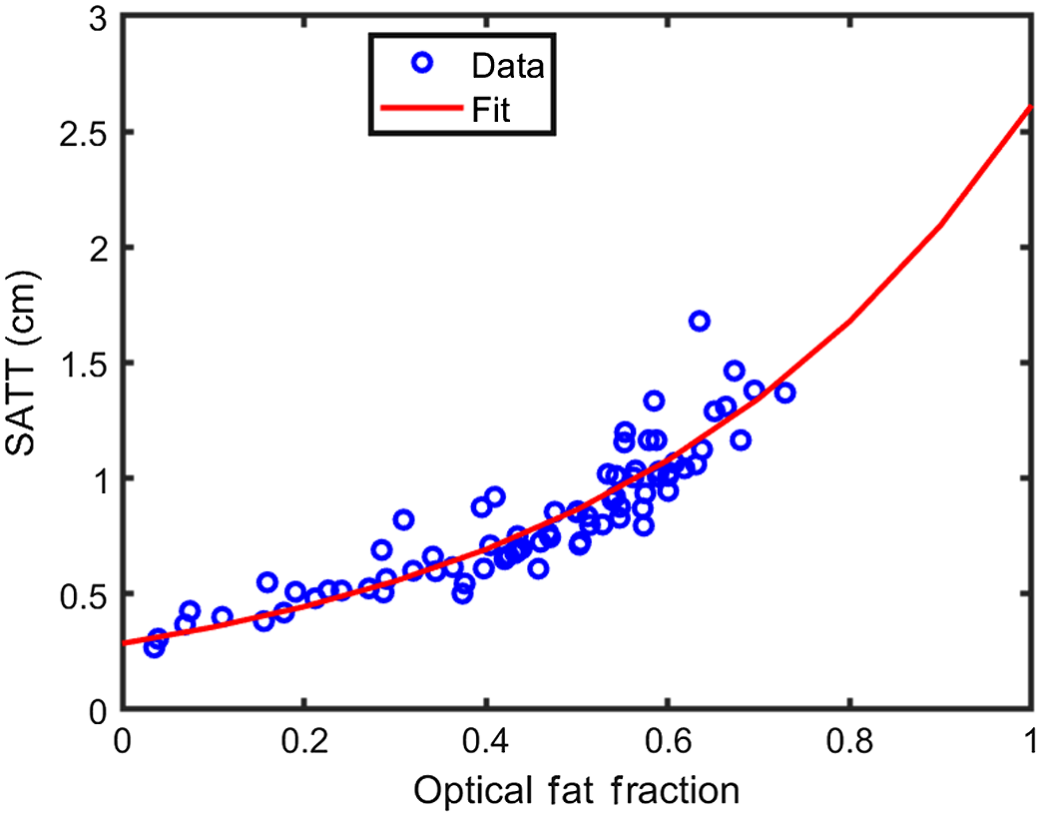
The relationship between OFF as calculated from DOSI data and SATT as measured with ultrasound. Data from 79 participants were collected from both the vastus lateralus and rectus femoris before being averaged together for each participant into a single value for the quadriceps. A single-term exponential function was fit to the data and is plotted in red. Fitted coefficients can be found in the text.

**Fig. 5:**
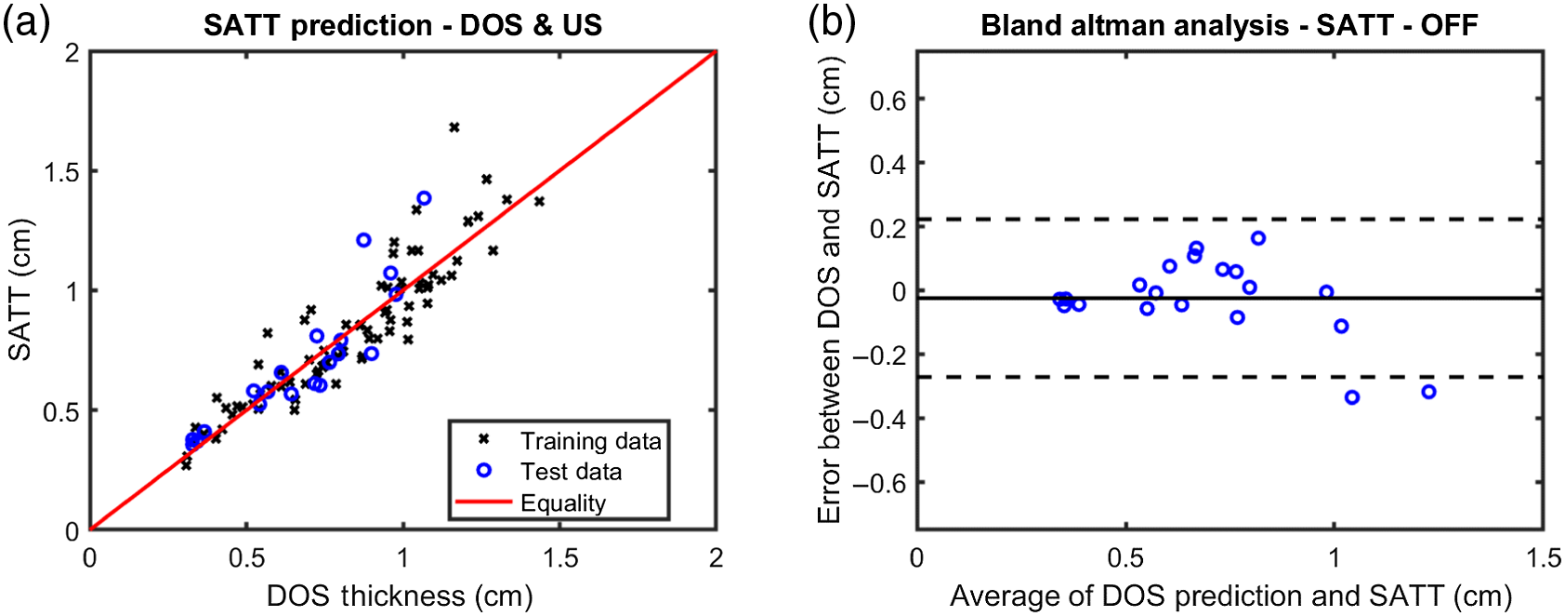
The ability of OFF from DOSI to predict SATT as measured by ultrasound. (a) 20 participants (blue dots) were not included in the initial exponential curve fitting (black Xs) and were used here to evaluate predictive accuracy. SATT as predicted by DOSI data is plotted against measured SATT values from ultrasound. A red line is shown to indicate perfect agreement. (b) A Bland–Altman plot is shown to better visualize error in the predictive method. The x-axis shows the average between DOSI and ultrasound SATT values while the y-axis shows the difference between the two SATT values. The black solid line shows the mean error of −.02  cm, while the two dotted lines indicate a 95% confidence interval (±1.96 standard deviations) for the differences of −0.27 to 0.22 cm.

Using data from 79 participants, OFF data was fitted against SATT given an assumed functional form of

$$\mathrm{SATT}=ae^{b.\mathrm{OFF}},$$ (8)

where a and b were free parameters (Fig. 4). After optimization in MATLAB’s curve fitting app, a and b were determined to be 0.2856 and 2.214, respectively. Data from the 20 participants excluded during fitting were input into the optimized function to predict SATT [Figs. 5(a) and 5(b)]. Predictions had a mean error with respect to actual SATT of −0.02±0.13  cm (mean±standard deviation). Predictions lost accuracy as SATT increased [Figs. 5(a) and 5(b)].

### 3.3 Fat and Lean Tissue Distinction by DOSI

**Fig. 6:**
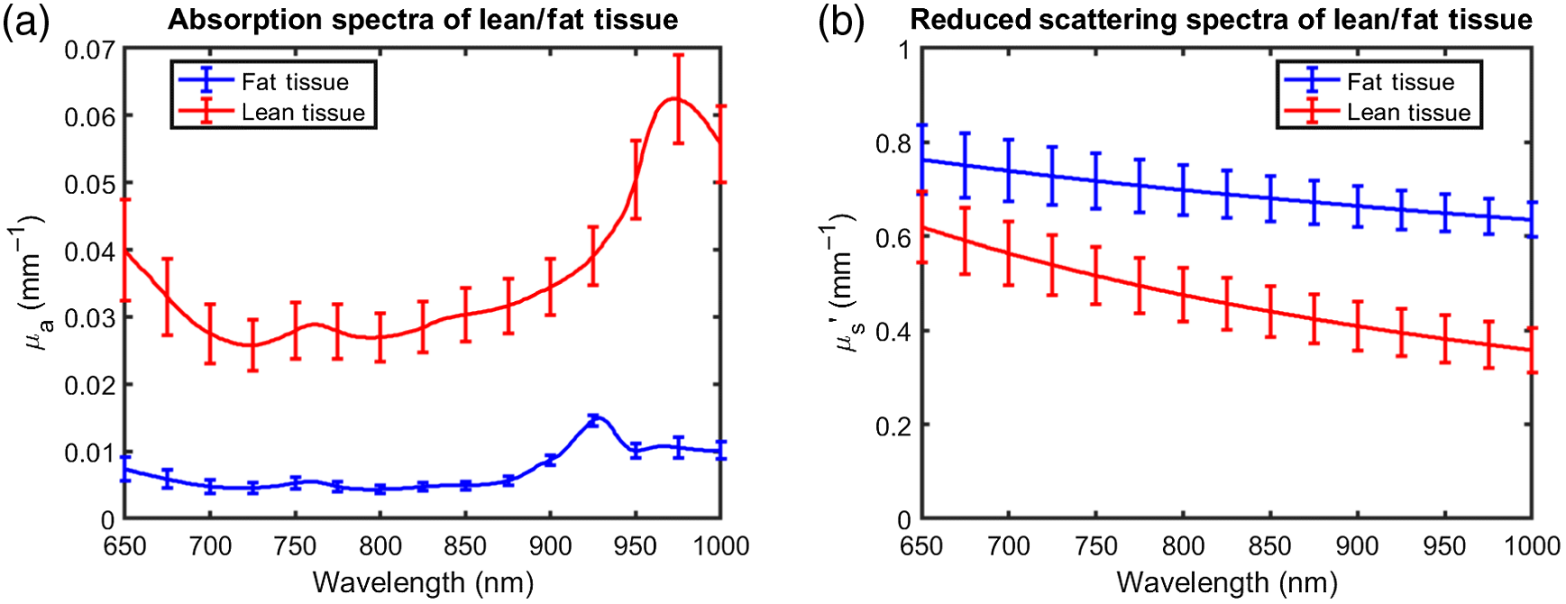
Optical properties for fat tissue and lean tissue measured from rectus femoris and vastus lateralus, respectively. Fat tissue measurements were selected by finding the five participants with thickest SATT values as measured by ultrasound (SATT=1.55±0.14  cm). Similarly, lean tissue measurements were selected by finding the five participants with thinnest SATT values as measured by ultrasound (SATT=0.25±0.02  cm). Each line is the average of data from five participants with error bars representing standard deviations.

In general, SATT was greater at the RF location (0.88±0.30  cm) than the VL location (0.71±0.30  cm). In addition, the overall thickest SATT measurements came from the RF while the thinnest SATT measurements from the VL. We isolated optical properties (absorption and reduced scattering coefficients) and optical chromophore concentrations from the RF of the five participants with thickest RF SATT (SATT≈1.55±0.14  cm) and from the VL of the five participants with the thinnest VL SATT (SATT≈0.25±0.02  cm) [Figs. 6(a) and 6(b) and Table 3]. These two sets of data approximate non-invasive diffuse optical measurements where the signal is predominantly from either adipose tissue or muscle tissue. The overall levels of absorption in lean tissue are 500% to 600% higher than fat tissue at all wavelengths except 850 to 950 nm where the fat absorption peak is strongly absorbing enough to keep levels of lean tissue absorption to ∼300% higher. Alternatively, reduced scattering coefficients in fat tissue range from 20% to 80% higher than lean tissue.

**Table 3:** DOSI chromophore and scattering parameters for fat tissue (n=5) and lean tissue (n=5).

Parameter	Lean avg	Lean std. dev.	Fat avg	Fat std. dev.
SATT (cm)	0.25	0.02	1.55	0.14
Age (years)	22.9	8.3	12.5	2.9
OFF (unitless fraction from 0 to 1)	0.6	1.4	68.7	4.2
OLF (unitless fraction from 0 to 1)	99.4	1.4	31.3	4.2
THbMb (μMolar)	125.2	18.3	19.4	3.2
${\mathrm{StO}}_{2}$ (%)	67.1	3.7	63.0	6.7
Water (%)	73.0	8.1	11.3	3.3
Fat (%)	1.1	2.5	67.3	3.4
$A_{500}$ (${\mathrm{mm}}^{-1}$)	0.87	0.13	0.85	0.11
b (unitless)	−1.28	0.21	−0.42	0.10

Using US measurements of SATT from rectus femoris and vastus lateralus tissue, participants with the five thickest (fat tissue) and five thinnest (lean tissue) SATT values were identified, respectively. Results from each subgroup of five participants were averaged and are shown in this table.

In lean tissue, THbMb concentration was 125.2±18.3  μMolar, ${\mathrm{StO}}_{2}$ was 67.1±3.7%, tissue water content was 73.0±8.1%, tissue fat content was 1.1±2.5%, reduced scattering amplitude was $0.87\pm 0.13\;{\mathrm{mm}}^{-1}$, and reduced scattering power was −1.28±0.21. In addition, participants comprising the lean subcohort were aged 22.9±8.3  years old and had OFF and OLF values of 0.6±1.4% and 99.4±1.4, respectively. In fat tissue, THbMb concentration was 19.4±3.2  μMolar, ${\mathrm{StO}}_{2}$ was 63.0±6.7%, tissue water content was 11.3±3.3%, tissue fat content was 67.3±3.4%, reduced scattering amplitude was $0.85\pm 0.11\;{\mathrm{mm}}^{-1}$, and reduced scattering power was −0.42±0.10. Participants comprising the fat subcohort were aged 12.5±2.9  years old and had OFF and OLF values of 68.7±4.2% and 31.3±4.2, respectively.

### 3.4 Whole-Body DOSI and Dual-Energy X-Ray Absorptiometry

**Fig. 7:**
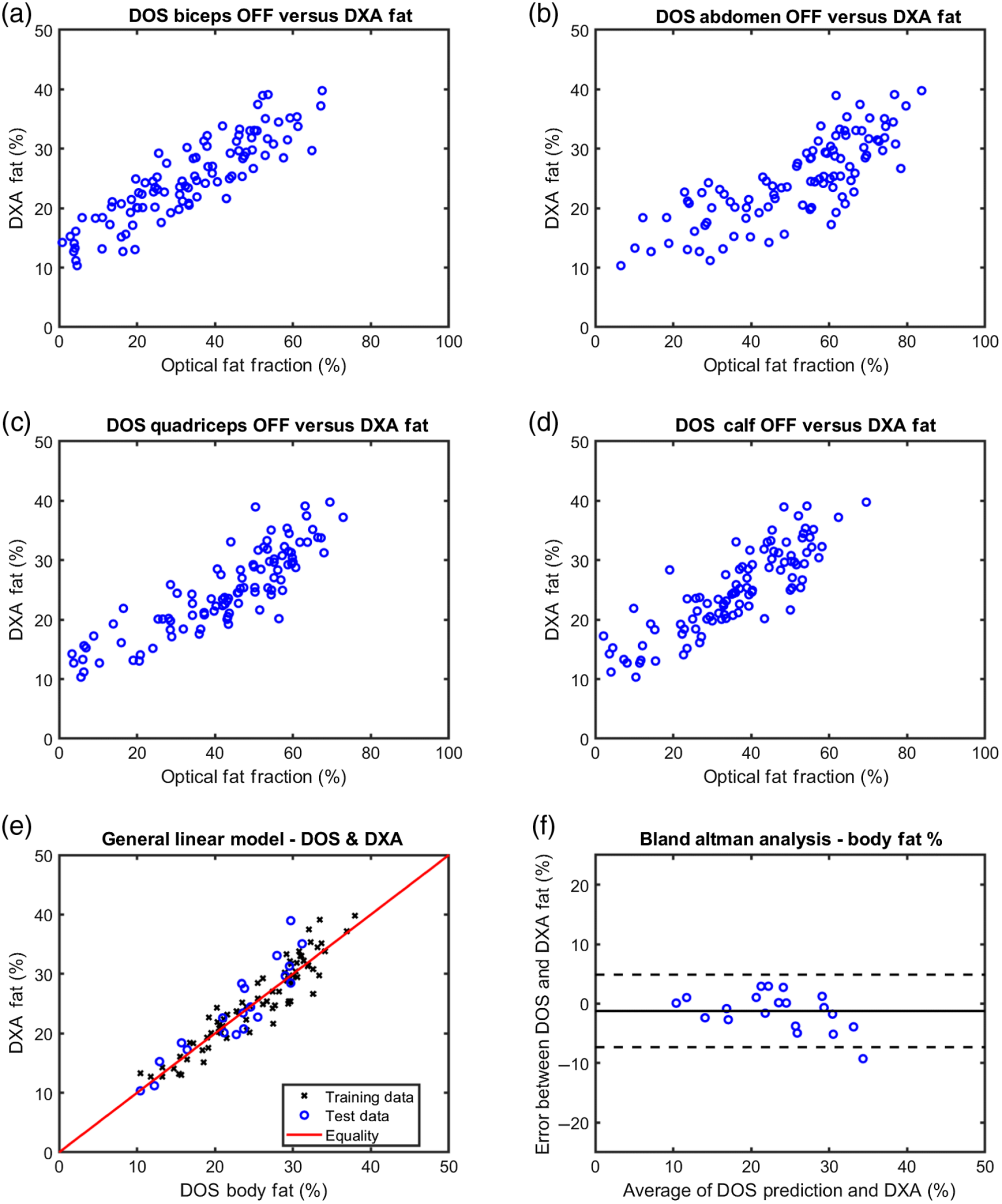
DOSI optical fat fraction results for each of the four general anatomical regions compared to whole-body fat% according to DXA, as well as a generalized linear regression model and predictive analysis. (a–d) OFF results from the biceps, abdomen, quadriceps, and calf, respectively, plotted against whole-body fat% from DXA for all 99 participants. (e) Results from 79 participants (black X marks) which were used in a generalized linear regression model and 20 participants which were left out of the model (blue circles). The trained model was used to evaluate data from the 20 excluded participants and resulting predictions are shown. (f) A Bland–Altman analysis is shown for the 20 participants excluded from the linear regression modeling. The x-axis shows an average between the measured DXA fat% and the predicted DOSI fat% while the y-axis shows the difference between the measured and predicted results. The black solid line shows the mean error of −1.2%, while the two dotted lines indicate a 95% confidence interval (±1.96 standard deviations) for the differences of −7.3% to 4.9%.

Significant correlations were detected between DOSI OFF at each general tissue location and whole body DXA fat% [Figs. 7(a)–7(d)]. In addition, nearly identical correlations were detected between DOSI OLF at each general tissue location and whole body DXA LST%. These figures are not presented, but the relationship can be inferred from the OFF/fat% relationship since OFF versus OLF and fat% versus LST% are directly inversely related. As OFF/OLF from all general anatomical regions showed significant associations with whole-body DXA measures, all four locations were included in the generalized linear regression models to predict DXA measures. For each model, the same 79 participants were randomly selected for linear regression while the remaining 20 were left out to assess predictive accuracy.

For prediction of DXA fat%, OFF resulted in a mean error of −1.2% with a 95% confidence interval between −7.3% and 4.9% [Figs. 7(e) and 7(f)]. For prediction of DXA LST%, OLF resulted in a mean error of 1.2% with a 95% confidence interval between −4.7% and 7.1% (figures not shown for reasons described in the previous paragraph).

## 4 Discussion

In this study, DOSI was used to determine both individual tissue composition and whole-body composition in a large cohort of participants ranging in age from 7 to 34 years old. These optical results were compared with US measurements of SATT and DXA assessments of body composition, leading to our proposal for two new optical metrics: the OFF and OLF, which utilize DOSI outputs to characterize the adiposity/leanness of tissue. This study adds to previous literature, which collectively validates measurements from DOSI in humans by placing optical parameters in context of traditional and more commonly used modalities in today’s medical practices.^9^^,^^13^^,^^23^ In addition, this study characterizes optical properties (absorption and scattering behavior) in lean and fat tissue, which can be used by the diffuse optics research community. Finally, methods were proposed to predict SATT from a single DOSI measurement and whole-body fat percentage (fat%) and lean-soft tissue percentage (LST%) given multiple DOSI measurements taken across the human body.

While significant correlations were detected between SATT and most DOSI parameters, the associations with ${\mathrm{StO}}_{2}$, b, and $\mu_{s}^{\prime }(800\;\mathrm{nm})$ were weak ($R^{2}<0.5$), suggesting that only a small amount of variance in SATT could be explained by these three DOSI parameters. Associations were stronger between SATT and THbMb, water, and fat, so these parameters were used in the definition of two complementary parameters: OFF and OLF. These two parameters are inversely related, sum to 100%, and were designed to describe the level of adiposity or leanness, respectively, of a tissue. We assumed an exponential relationship between OFF and SATT and used data to fit a simple exponential function. This fitted exponential was used to predict SATT in a subset of 20 participants. We did not explore other functions that might more accurately describe the relationship between OFF and SATT. While predictions had minimal error (1 to 2 mm) when SATT was less than 1 cm, predictions lost accuracy as SATT surpassed 1 cm suggesting a sensitivity limit with DOSI. This finding was expected with a fixed source-detector separation of 28 mm and our rough sensitivity limit seems to be ∼1/3 source-detector separation, which matches findings from other researchers that report a “rule-of-thumb” for optical penetration depth as a function of source-detector separation.^24^ In future work, this must be considered when working with obese patients or when measuring tissues with SATT>1  cm (e.g., abdomen). In addition, it is important to note that the sensitivity to adiposity as discussed in this study is different than, for example, as discussed in Ganesan et al.^9^ In the work of Ganesan et al., the study was designed to isolate adipose tissue and to avoid potential contamination by the underlying muscle or visceral organs. In the current study, contamination by underlying muscle tissue was expected and is the primary cause of a decreased “adipose” signal. The layered nature of human tissue is what allows for the DOSI OFF assessment to predict SATT. If the layered structure is significantly different, such as on the head of a person, where there is very little adipose or muscle between skin and the skull, then the OFF parameter will no longer be predictive of an upper layer thickness. This could also be important to consider in conditions where the adipose or muscle tissues are diseased or altered by some abnormal condition. For example, if an individual’s muscle tissue contains a large amount of intramuscular fat, then OFF would overestimate the upper SATT, even though it correctly reflects the overall adiposity of the tissue that was measured. In summary, adipose layer thickness prediction by OFF as described in this study has at least two limitations: (1) depth sensitivity, which can be overcome by modifications to hardware to increase source-detector separation and (2) application to expected anatomical sites with layered adipose and muscle.

We isolated optical properties and derived optical parameters from the participants with the thickest SATT and the thinnest SATT. While this information adds to a growing body of reference literature for optical properties of human tissue,^9^^,^^13^^,^^25^ the age range of participants that these data represent is unique compared to previous studies. As expected, absorption coefficients were much higher in participants with thinner SATT than in those with thick SATT (Fig. 6). In addition, the relative magnitude of the fat absorption peak (∼930  nm) versus the water absorption peak (∼970  nm) is a clear feature of thick versus thin SATT. Finally, participants with thin SATT presented lower scattering coefficients but a steeper wavelength dependence (Fig. 6). In the subset of participants with thick SATT, ${\mathrm{StO}}_{2}$ and fat results were comparable to those from Ganesan et al. (63.0% versus 60.0% and 67.3% versus 64.3%, respectively), while the THbMb, water, scattering amplitude, and scattering power were in disagreement.^9^ Not only were the participants from the current study much younger (16 years old) than those from Ganesan et al.’s study (56 years old), but the individuals in Ganesan et al.’s study were obese and known to have some presentations of metabolic syndrome. This finding suggests that there could be age-related differences in adiposity that are insufficiently described by standard measures of oxygen saturation (${\mathrm{StO}}_{2}$) or adiposity (fat). Scattering parameters in particular have been of high interest in relation to lipid content and this finding suggests they could be useful in distinguishing types of fat (or age-related alterations to fat). In participants with thin SATT, both optical properties and derived parameters are on the lean end of the range presented in our previous work studying calf muscles.^13^ US was not used to quantify SATT in our previous work, but individuals from that study with the highest concentrations of THbMb showed ∼120  μMolar while the participants with thinnest SATT in this current study showed 125.2  μMolar.

Prediction of fat% and LST% using OFF and OLF, respectively, showed a minimal mean error (−1.2%) with a 95% confidence interval spanning from −7.3% to 4.9%. The largest errors came in individuals with high fat% with DOSI underestimating fat% by ∼10% in one case. This error was expected in participants with higher fat% for the same reasons that SATT predictive accuracy decreased with increasing SATT, namely that the fixed source-detector separation of DOSI limits the tissue penetration depth (∼1  cm in this study). For individuals with >30% fat%, we predict that most of the tissues measured with DOSI (biceps, abdomen, quadriceps, and calf) would have had 1 cm or greater SATT. By increasing the source-detector separation of DOSI, greater predictive accuracy would likely be achieved in populations that are more obese. The tradeoff with increased source-detector separation, however, is a decreased signal-to-noise ratio, which would be most apparent in measurements of high absorbing tissues (lean tissue). Given the relatively high predictive accuracy of SATT and fat% in individuals with thin SATT and low fat%, it seems likely that the overall predictive range could be extended with an increased source-detector separation to ∼35  mm. In addition, we likely lost some information content in reducing the size of our data set by averaging data in measurement locations of close proximity. We hypothesized that measurement locations of close proximity would share similar tissue composition, and while this was true in some cases, it was incorrect in others, such as the VL and RF from the quadriceps as discussed in Sec. 3.3. This could be mitigated in future work by leveraging more advanced statistical methods that account for repeated measures.

While we sampled across males and females, younger and older individuals, and BMI (as examples), we did not power the study to perform sub-cohort analyses and are unable to make accurate statements on one group compared with another. As such, the analyses in this study are focused on highlighting feasibility across the overall cohort that contains a large range of different types of participants. We expect that future studies will carefully design their participant demographics to answer specific questions of clinical interest. For example, there are age-dependent changes in intramuscular fat, and it would be interesting to recruit the correct participants to understand and validate the performance of DOSI across age. In addition to being limited in sample size, we also were unable to recruit obese adults into this study. An interesting extension to this work would be to expand the range of adiposity in the adult sub-cohort and determine if the preliminary relationships shown here (i.e., DOS versus US and DOS versus DXA) are age-dependent.

In summary, we showed that DOSI can determine site-specific and body-wide levels of adiposity and leanness, and we proposed two new DOSI-derived metrics to encapsulate such assessments: the OFF and the OLF. We also provide reference optical properties from lean and fatty tissues and DOSI-derived parameters across a young population (7 to 34 years) and a wide range of tissue sites. These results will be useful as NIRS technologies mature and produce clinical products beyond pulse and tissue oximeters. Future studies should analyze additional populations, including those with disease, to better elucidate the applications of DOSI for site-specific and body-wide composition analysis.

## Code, Data, and Materials Availability

Analysis software built in MATLAB is maintained by the research team and can be made available by request from the corresponding author. In addition, the de-identified NIRS datasets acquired in this study can also be made available by request from the corresponding author.
